# A Novel Index for Prompt Prediction of Severity in Patients with Unstable Angina Pectoris

**DOI:** 10.1155/2020/7651610

**Published:** 2020-01-03

**Authors:** Mustafa Bolatkale, Ahmet Cagdas Acara

**Affiliations:** ^1^Medipol University Hospital, Department of Emergency Medicine, Istanbul, Turkey; ^2^Bozyaka Research and Training Hospital, Department of Emergency Medicine, Izmir, Turkey

## Abstract

**Objectives:**

Rapid risk stratification by emergency department (ED) physicians to evaluate patients with chest pain for predicting the short-term occurrence of major adverse cardiac event (MACE) is crucial. The aim of this study was to investigate the predictive value of platelet-lymphocyte ratio (PLR) levels and compare with the modified heart score (m-HS) and stress testing to predict the severity of high-risk patients with unstable angina pectoris (UAP) in the ED.

**Methods:**

This study is prospective which included 316 patients with UAP and 316 control healthy subjects. The study took place from 01 April 2016, until 01 April 2017, in Medipol University.

**Result:**

The mean PLR levels in the UAP group were higher than those in the control group (*p* < 0.001). The mean PLR of the m-HS ≥4 group was higher than that in the m-HS ≤3 group (*p* < 0.001). The mean levels of PLR in the subgroups based on the stress testing positivity were higher than those in the stress testing negative subgroup (*p* < 0.001). PLR levels were positively correlated with the m-HS, stress testing, and treatment decision in this study (*r* = 0.559; *p* < 0.001; *r* = 0.582; *p* < 0.001; *r* = 0.789; *p* < 0.001, respectively).

**Conclusion:**

A positive correlation was determined with an increase in m-HS, treatment decision, and positive exercise testing as the PLR levels increased, indicating the severity of high risk of UAP in the ED.

## 1. Introduction

Acute coronary syndrome (ACS) is the leading cause of death worldwide. ACS covers a wide spectrum of clinical conditions, ranging from unstable angina pectoris (UAP) to non-ST elevation myocardial infarction (N-STEMI) or ST elevation myocardial infarction (STEMI). UAP and NSTEMI are closely related conditions, with similar pathophysiological origins and clinical presentations, but they differ in severity. In a patient experiencing UAP, no biomarker can be detected in the bloodstream hours after the initial onset of ischemic chest pain [1]. Although only white clots are found in patients with UAP/NSTEMI, red clots form in patients with STEMI [1]. UAP is considered under the umbrella term of ACS, in which patients are admitted with acute chest pain or shortness of breath, with neither ST elevation nor abnormal cardiac enzymes [2]. UAP is characterized by <50% atherosclerotic plaque, which is not expected to obstruct coronary blood flow. Annually, UAP causes more than 1 million hospital admissions [3]. However, normal values of troponin (Tn), a normal electrocardiogram (ECG), and negative stress test still do not exclude ACS completely [4]. It is important to determine whether patients have ACS, as any delay in diagnosis and treatment can have a negative impact on the prognosis. A missed MI due to unrecognized UAP continues to be one of the most common reasons behind malpractice lawsuits against physicians, comprising nearly 20% of all claims [5]. If the patient remains pain-free and the results of ECG and cardiac marker tests are negative, an early stress test should be performed either before discharge or on an outpatient basis within 72 hours in patients with UAP [1]. The sensitivity and specificity of the stress test for a single vessel are 68% and 77%, respectively. In patients with multivessel coronary artery disease, sensitivity is 81% and specificity is 66% [6]. Rates of false positive exercise electrocardiography stress test in female patients has been reported to be 18.7%, with the most common predisposing causes known to be coronary artery disease, hypertension, and left ventricular hypertrophy [7].

A significant proportion of patients with chest pain undergo advanced medical evaluation during visits, resulting in longer and more costly ED stays. Many patients presenting with chest pain are currently hospitalized and extensively evaluated with noninvasive stress testing or imaging or with invasive coronary angiography (CAG) [2]. The gold standard diagnostic test for ACS is CAG, but this cannot be applied to all cases of chest pain due to UAP in the ED [4].

There are many risk stratification scores for ACS, but with the exception of the modified-heart score (m-HS), scoring systems are time-consuming and can delay treatment in patients with ACS caused by myocardial damage due to low ejection fraction. M-HS is a rapid risk stratification tool, designed by ED physicians to evaluate patients with chest pain according to their short-term risk of major adverse cardiac event (MACE, defined as acute myocardial infarction (AMI), need for percutaneous coronary intervention (PCI) or coronary artery bypass graft surgery (CABG), and death within 6 weeks) to help identify low-risk patients, suitable for earlier ED discharge within 30 days of index ED visit [8, 9]. However, the original HS included cTn levels in the score in a way that allowed a patient with an elevated cTn to be considered low-risk [8]. Generally, high-risk patients are admitted to the cardiology or medical wards and those at low risk are managed in an observation unit or sent home.

In the pathophysiology of ACS, inflammatory mediators and endothelial dysfunction play a pivotal role in the coronary inflammation found during UAP [10]. Platelets play a key role in the pathophysiology of ACS. Higher levels of inflammatory markers are associated with the severity of coronary artery disease (CAD) and worse cardiovascular outcome [11]. The platelet-lymphocyte ratio (PLR), which is calculated by the division of absolute platelet count by absolute lymphocyte count, has been stated to be a new indicator of inflammatory response. Inflammation leading to ACS has encouraged research into the clinical usage of new inflammatory biomarkers [9]. The PLR has been claimed to be a marker to help identify thrombotic activity and inflammation in cardiac diseases [12].

Early diagnosis and the initiation of appropriate treatment within 24 hours are essential to reduce UAP-related mortality and morbidity [13]. If patients at high risk of UAP could be recognized early in the diagnostic process, mortality, morbidity, and negative effects of delayed treatment could be potentially reduced and a good outcome could be provided with cost-effective treatments [10]. Therefore, there is still a need for a sensitive, specific marker for the prediction of severity in patients with UAP.

Recent studies have shown that PLR, which is evaluated as a prognostic marker of ACS, may also be increased in patients with N-STEMI and STEMI [14, 15]. Previous studies have revealed that PLR levels are significantly increased in MI, stroke, and subsequent heart failure [16]. However, there are no studies regarding PLR levels in patients with UAP and no studies which have examined PLR levels compared with m-HS and stress testing to predict the severity and make the treatment decision in patients with UAP.

The aim of the present study was to compare PLR levels with m-HS and stress testing in the prediction of severity and treatment decision in patients with UAP.

## 2. Methods

Approval for this prospective study was granted by the Medipol University Clinical Research Ethics Committee (decision no: 11.03.2016/E.3931-145). This prospective study was conducted in the Medipol University Hospital Adult ED. The study took place from 01 April 2016, until 01 April 2017. All patients provided signed informed consent. The study was performed in accordance with the Declaration of Helsinki.

### 2.1. Study and Control Groups

Our study group was comprised of adult patients with a diagnosis of UAP diagnosed in the ED. The control group was comprised of healthy adults with no acute or chronic disease. Patients were excluded if they had a previously known disease which would explain the chest pain other than angina or if they did not agree to participate in the study. All patients in both groups were ≥18 years old. The groups were classified as “UAP” and “Control.” Gender, age, and PLR levels were recorded for both groups, and the latter was examined in the UAP group: blood urea nitrogen (BUN), creatinine, hemograms, CK-MB, 0 and 3-hour cTnI, m-HS, stress testing results, and treatment decision. The patients with UAP were separated into 3 subgroups, based on low (≤3) and high (≥4) m-HS [4], positive and negative stress testing, and the treatment decision of CAG (only conventional medical treatment), PCI, and CABG.

### 2.2. Biochemical Analysis

All blood samples were taken from the brachial veins. Creatinine and BUN were measured (Cobas 6000 auto analyzer, Roche, Tokyo, Japan). Blood samples for hemograms were collected in 2 ml ethylenediaminetetraacetic acid (EDTA) tubes and analyzed using an automated hematology analyzer (XT-2000I; Symex, Osaka, Japan). The PLR levels were calculated as the ratio of platelet count to lymphocyte count. Blood samples were centrifuged within 30 minutes and CK-MB, and 0- and 3-hour cTnI were determined using an AQT90 FLEX analyzer device (Radiometer, Copenhagen, Denmark) with the immunoassay method. Cutoff values for CK-MB and cTnI were 7.2 *μ*g/L and 0.023 *μ*g/L, respectively.

### 2.3. m-HS and Stress Testing

The m-HS was prospectively applied to the population in the ED. The acronym m-HS is an abbreviation of the four parameters evaluated [5]: history (highly suspicious—2 points; moderately suspicious—1 point; slightly suspicious—0 points), ECG (significant ST-depression—2 points; nonspecific repolarization disturbance—1 point; normal—0 points), age (≥65 years—2 points; 45–65 years—1 point; ≤45 years—0 points), risk factors for coronary heart disease (≥3 risk factors—2 points; 1 or 2 risk factors—1 point; no risk factors—0 points). The identified risk factors for coronary heart disease include prior acute MI, percutaneous coronary intervention or coronary artery bypass grafting surgery, hypertension, diabetes mellitus, smoking history, hyperlipidemia, and family history.

According to the total score applied, patients with UAP were separated as m-HS ≤3 low risk and m-HS ≥4 high risk of a MACE, within 6 weeks after presentation at the ED [8]. Lower scores (m-HS ≤ 3) led to a recommendation of early discharge, and high scores (HS ≥ 4) suggested clinical observation or performance of noninvasive investigations and admission for more invasive strategies [8]. Stress testing was applied to patients with UAP with m-HS ≤3. The stress testing was modified according to the Bruce protocol [17].

### 2.4. Statistical Analysis

Statistical analyses of the data obtained in the study were made using SPSS 25.0 software (SPSS Inc., Chicago, IL, USA). Variables were stated as mean ± standard deviation or median with interquartile range. Student's *t* test was used to compare mean values, and the Mann–Whitney *U* test was used to compare median values. Frequencies were compared with the chi-squared and Fisher's exact tests. Spearman's correlation tests were applied for correlation analyses. The median and mean PLR values were calculated. To determine a cutoff value of PLR level for UAP, receiver operating characteristic (ROC) analysis was performed in sensitivity and specificity calculations. Receiver-operating characteristic analyses were used to detect the cutoff value of PLR in the prediction of low to high m-HS. The PLR levels were compared between the subgroups based on the m- HS, stress testing, and treatments using one-way analysis of variance (ANOVA) testing with least significant difference (LSD) post hoc analysis. A value of *p* < 0.05 was considered statistically significant.

## 3. Results

During the study period, a total of 14,876 patients were admitted to the ED. After application of the exclusion criteria, 316 patients in the ED with UAP were included as the study population. The study included 316 patients admitted with UAP and 316 control healthy subjects. The mean age was 64.68 ± 10.67 years in the UAP group and 63.89 ± 10.42 years in the control group (*p* = 0.346). The UAP group was comprised of 112 (35%) females and 204 (65%) males, and the control group was comprised of 118 (37%) females and 198 (63%) males (*p*=0.621). The mean platelet levels of the UAP group (241.43 ± 55.48 10^3^/*μ*L) were higher than those of the control group (221.58 ± 56.78 10^3^/*μ*L) (*p* < 0.001). The mean lymphocyte levels of the UAP group (2.25 ± 0.87 10^3^/*μ*L) were higher than those of the control group (2.12 ± 0.51 10^3^/*μ*L) (*p* : 0.025).

The mean lymphocyte levels of the UAP group were 2.25 ± 0.87 10^3^/*μ*L. In the control group, the mean platelet levels were 221.58 ± 56.78 10^3^/*μ*L and lymphocyte levels were 2.12 ± 0.51 10^3^/*μ*L. The mean PLR levels of the UAP group (130.09 ± 48.52) were higher than those of the control group (99.01 ± 21.51) (*p* < 0.001).

**Figure 1:**
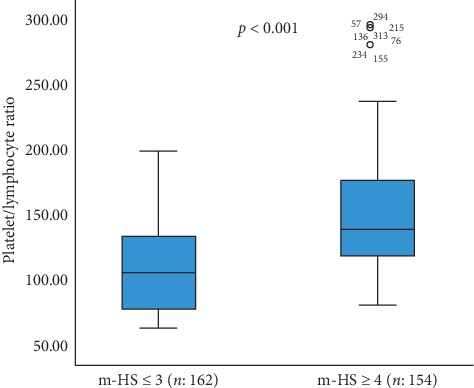
Distribution of PLR levels for m-HS ≤3 and m-HS ≥4 subgroups (PLR: platelet-lymphocyte ratio; m-HS: modified-heart score). PLR levels were determined to be positively correlated with m-HS (*r* = 0.559; *p* < 0.001, Spearman's).

**Figure 2:**
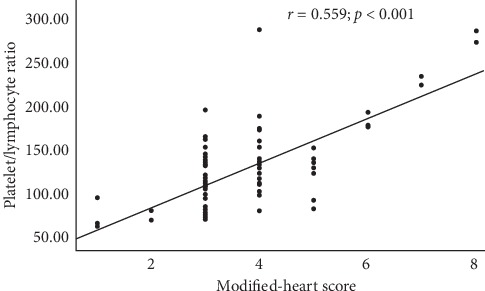
Correlation between PLR levels and m-HS (PLR: platelet-lymphocyte ratio; m-HS: modified-heart score).

The area under the ROC curve for PLR level was 0.713 (95% confidence interval [CI], 0.672–0.753), and the PLR level had sensitivity of 58.2% and specificity of 80.4% at 117.99. When the m-HS of the 316 patients in the study were examined, 154 patients were evaluated with m-HS of ≥4 and 162 patients with m-HS ≤3. The mean PLR of the m-HS ≥4 group (*n*: 154) was higher than that of the m-HS ≤ 3 group (*n*: 162) (152.76 ± 52.25 vs. 108.54 ± 32.46; *p* < 0.001) (Figures 1 and 2).

The area under the ROC curve for PLR level in the UAP subgroups based on m-HS, between m-HS ≤3 (low) and m-HS of ≥4 (high), was 0.770 (95% confidence interval (CI), 0.720–0.821). The PLR level had sensitivity of 71.4% and specificity of 74.1% at 125.40.

**Figure 3:**
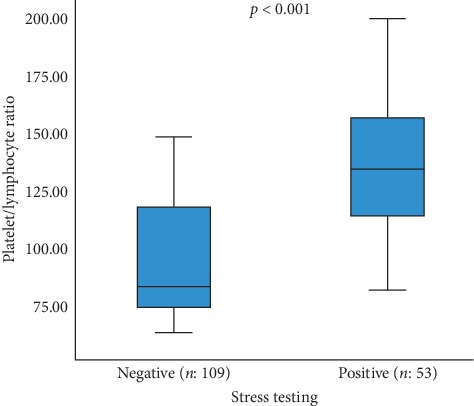
Distribution of PLR levels for each subgroup according to exercise testing (PLR: platelet-lymphocyte ratio).

Exercise testing was applied to 162 patients with m-HS ≤3. Of these, 53 had positive exercise testing output, 30 patients underwent PCI, 4 patients underwent CABG, and 19 patients had taken only conventional medical treatment. The mean levels of PLR in the subgroups based on the stress testing positivity (135.55 ± 30.08) were higher than in the stress testing negative subgroup (95.40 ± 25.57) (*p* < 0.001) (Figure 3). The mean levels of PLR in the subgroups based on the stress testing positivity with significant CAD (*n*: 34; 151.47 ± 23.87) were higher than those in the stress testing positivity without significant CAD subgroup (*n*: 19; 107.06 ± 15.27) (*p* < 0.001).

**Figure 4:**
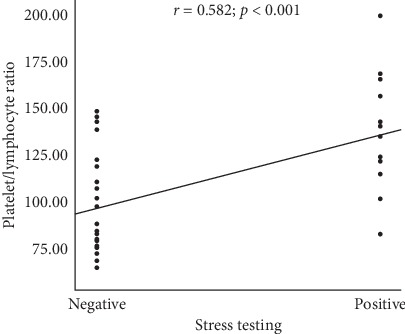
Correlation between PLR levels and stress testing (PLR: platelet-lymphocyte ratio).

PLR levels were positively correlated with stress testing in this study (*r* = 0.582; *p* < 0.001, Figure 4).

The area under the ROC curve for PLR level in the UAP subgroups based on exercise testing to differentiate between stress testing abnormal and normal was 0.843 (95% confidence interval (CI), 0.782–0.903), and the PLR level was determined with sensitivity of 84.9% and specificity of 73.4% at 112.02.

**Figure 5:**
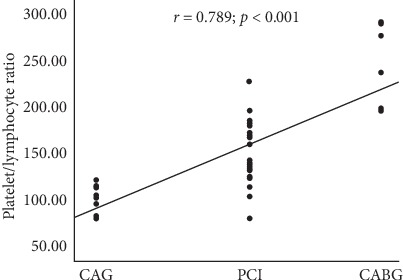
Correlation between PLR levels and treatment decision (PLR: platelet-lymphocyte ratio; CAG: coronary angiography; PCI: percutaneous coronary intervention; CABG: coronary artery bypass graft).

The PLR levels were statistically significantly different in the subgroups based on treatment decision (*p* < 0.001, ANOVA test). The mean PLR level was 103.95 ± 12.36 in the CAG subgroup (*n*: 48), 146.37 ± 26.69 in the PCI subgroup (n: 135), and 248.34 ± 41.00 in the CABG subgroup (n: 24) (*p* < 0.001). A positive correlation was determined between PLR levels and treatment decision in this study (*r* = 0.789; *p* < 0.001, Figure 5).

## 4. Discussion

In this study, PLR levels were compared with m-HS and stress testing in the prediction of low-high risk and treatment decision in patients with UAP in the ED. A positive correlation was determined with an increase in m-HS and positive exercise testing as the PLR levels increased, indicating the severity of high risk of UAP in the ED. The correlation between PLR levels and the treatment decision provides early information about patients admitted to the ED in respect of prognosis, and thus the decision of treatment for chest pain due to UAP can be guided. To the best of our knowledge, this is the first study to have demonstrated an association of PLR levels with the m-HS and the severity of coronary atherosclerosis in patients with UAP. According to the current study findings, higher PLR values on admission were significantly associated with high m-HS in patients with UAP.

Atherosclerosis is a complex and multifactorial inflammatory disease characterized by low-grade arterial inflammatory lesions that can develop through CAD progression, and inflammatory markers can be shown in circulation [18–20]. Inflammation plays a key role in the development, progression, and complications of atherosclerosis. Previous studies have demonstrated that higher platelet and lower lymphocyte counts are associated with MACE and poor clinical outcomes in various cardiovascular diseases [17, 21]. Platelets play a major role in the atherothrombotic process and pathogenesis of AMI, and an elevated platelet count is related to increased infarct size as well as short- and long-term worse prognosis in patients with AMI [22]. The CADILLAC study showed that the level of platelets (which does not affect the effectiveness of percutaneous interventions) is significantly correlated with the incidence of restenosis and stent thrombosis [16].

Low lymphocyte count is related with increased inflammation, and a low lymphocyte count is a worse prognostic marker in patients with CAD [22]. Lymphocytes are a major part of chronic inflammation in the atherosclerotic process and infiltrate the ischemic myocardium during AMI [22]. A lower lymphocyte count due to increased cortisol as a response to stress has been associated with high mortality rates in AMI [22]. It has been proposed that, in response to physiological stress during myocardial ischemia or infarction, there is a release of cortisol and catecholamine, redistribution of lymphocytes to lymphatic organs, and apoptosis, which lead to lymphopenia [21]. A high level of physiological stress means high levels of cortisol and catecholamine, which can be translated into a lower lymphocyte count.

PLR is calculated from systemic inflammatory markers as a predictor of poor cardiovascular outcomes [11]. Recent studies have demonstrated that the PLR is associated with MACE and that it is an independent marker of mortality in some oncological and cardiac diseases [12, 23, 24]. In a recent study, a high PLR was found to be correlated with the recurrence of MI, stroke, and subsequent heart failure [16]. However, its relationship with UAP severity is not yet known. In the present study, high PLR levels may be an indication of increased inflammatory response, which is related to the extent of inflammation, atherosclerosis, and thromboembolic states. In another recent study, the PLR level was reported to be independently associated with the GENSINI score together with white blood cell (WBC) count, age, and low high-density lipoprotein (HDL) in multivariate analysis [11].

The m-HS includes elements of the history, ECG, age, and risk factors [8]. However, in the original HS, cTn levels were considered in a way that allowed a patient with an elevated cTn to be evaluated as low-risk [8]. In a previous study, it was suggested that when normal serial cTn values are combined with the HS, a very low-risk patient population can be identified, which could be discharged from the ED without further testing [8]. Low-risk patients (a score of ≤3) have been reported to have a low (1.7%) MACE rate [9]. The majority of physicians deem a miss rate of <1% for MACE as acceptable in screening tools [9]. Furthermore, 2% of AMI cases are inadvertently discharged from the ED, leading to worse outcomes and medical-legal issues [8]. The majority of these patients undergo a period of observation involving serial cardiac markers and stress testing or cardiac imaging [8]. This practice leads to increased costs but also to ED overcrowding, which has been associated with worse outcomes [8]. McCord et al. reported that excluding acute MI with a 1-hour protocol using high-sensitivity cardiac troponin-T in patients with a low m-HS (≤3) identifies a low-risk group that might be able to be immediately discharged from the ED without further cardiac testing. Using such a risk-stratification strategy is likely to have a greater impact in the US. Patients with a higher risk of m-HS (≥4) require cardiac troponin testing over a longer period of time [8]. In the present study, the mean levels of PLR of the m-HS ≥4 group were significantly higher than those of the m-HS ≤3 group. Early prediction with the diagnostic value of the PLR may provide extra time for the early treatment decision in patients at high risk of UAP. The correlation shown between PLR and m-HS could lead to early identification and prognosis.

Clinical algorithms can identify lower risk patients who can be safely further risk stratified using exercise testing [25]. In a series of 1,000 patients admitted to the ED with symptomatic low-risk chest pain, probably of cardiac origin, positive exercise test results were reported in 13% [25]. Khare et al. studied a lower risk population of 1,194 patients admitted to the ED because of chest pain and reported exercise testing results as positive in 9% and negative in 91% [26]. In another previous study, patients with positive exercise testing results underwent cardiac catheterization, and only 27% had significant obstructive coronary artery disease [26]. Patients in the current study determined with positive exercise testing underwent PCI and CABG, and only 21% had significant obstructive coronary artery disease.

The recently published, randomized TIMACS (Timing of Intervention in Patients With Acute Coronary Syndromes) trial compared the outcomes achieved with an early invasive strategy (intervention within 24 hours of presentation) and a delayed invasive strategy (intervention at any time >36 hours after presentation) in 3,031 high-risk patients with UAP/NSTEMI [1]. With the exception of high-risk patients with a Grace (Global Registry of Acute Coronary Events) risk score >140, the early invasive strategy was not seen to be superior to the delayed invasive strategy in reducing the primary end point of death, MI, or stroke at 6 months [1].

Antiplatelet and anticoagulant medication given to patients during ACS and PCI have significantly reduced the reinfarction and lowered the mortality rates [27]. These drugs are administered in catheter laboratories for emergency PCI, but in cases not treated due to operation failure or inappropriate anatomy and in those for whom an urgent CABG decision is made before the PCI procedure, these drugs can significantly increase the bleeding and hemorrhagic mortality, with the exception of cangrelor [28, 29]. Although urgent CABG surgery is recommended, the operation of these patients is delayed in many centers, and because of prolonged waiting time for CABG, the mortality rates increased in patients with CAD [30]. The results of the current study demonstrating the correlation of high m-HS and positive stress testing with high PLR levels have shown that there is a high likelihood that in cases of CAD referred for CA are preferential candidates for interventional therapies such as PCI and CABG. The bleeding and mortality rates may be reduced in association with antiplatelet and anticoagulant doses, which can be established through early consultation with cardiovascular surgeons before applying CAG.

Early diagnosis and the initiation of appropriate treatment within 24 hours are essential to reduce UAP-related mortality and morbidity rates [13]. This early prediction of severity and ischemia using a biomarker is important as it may improve the risk stratification of UAP patients, guide treatment decisions, and preserve patients from MACE and the risk of sudden cardiac death. Furthermore, this study has demonstrated that an increased PLR level on admission is an independent predictor of high risk for UAP. The results of the study have shown that PLR indices are sufficient to show high risk in patients with UAP and have a strong positive correlation to m-HS ≥4. Thus, the study hypothesis confirmed that PLR may be useful as a predictive biomarker for UAP because it can indicate the severity of CAD in patients with UAP. If these results are confirmed by further studies, the use of PLR may improve current predictive, prognostic strategies, and guide treatment decisions in patients with UAP in the ED.

## 5. Conclusion

In the present study, blood PLR levels were observed to be significantly increased in adult patients with UAP compared to healthy control subjects, and a strong positive correlation was determined between PLR and m-HS scores. Higher PLR may indicate an increased inflammatory response, which is related to the severity of coronary atherosclerosis in patients with UAP and this could therefore be a part of cardiovascular evaluation in the ED before further examination and treatment decision. PLR appears to be a cost-effective and practical tool predicting the severity of coronary atherosclerosis in patients with UAP. A positive correlation was determined with an increase in m-HS, treatment decision, and positive exercise testing as the PLR levels increased, indicating the severity of high risk of UAP in the ED.

## 6. Study Limitations

PLR may be affected by several pathological variables. However, it was not possible in this study to control all the variables that could influence PLR levels. There was no comparison of other inflammatory markers in the prediction of severity of UAP. The contact time to hospital was accepted as the initial hour for the purposes of this study. The period which started after the collection of the first blood samples was not standardized in every patient, and the number of subjects included in the study was limited.

## Data Availability

The data used to support the findings of this study are available from the corresponding author upon request.
